# Role of pelvicalyceal anatomy in the outcomes of retrograde intrarenal surgery (RIRS) for lower pole stones: outcomes with a systematic review of literature

**DOI:** 10.1007/s00240-019-01150-0

**Published:** 2019-08-01

**Authors:** Sulaiman Sadaf Karim, Luke Hanna, Robert Geraghty, Bhaskar K. Somani

**Affiliations:** Department of Urology, University Hospital Southampton NHS Foundation Trust, Southampton, SO16 6YD UK

**Keywords:** Flexible ureteroscopy, Infundibular pelvic angle, Infundibular width, Infundibular length, Pelvicalyceal anatomy, RIRS

## Abstract

Controversies exist on the influence of lower pole anatomy (infundibular pelvic angle, IPA; infundibular length, IL; and infundibular width, IW) for success and outcomes related to the treatment of stones in the lower pole. We wanted to look at the role of lower pole anatomy to study clinical outcomes in patients treated for isolated lower pole stones (LPS) using retrograde intra renal surgery (RIRS), and also perform a review to look at the published literature on the influence of pelvicalyceal anatomy on success with RIRS. Data were prospectively collected (June 2013–June 2016) for all patients who underwent RIRS for LPS, and the imaging was then retrospectively reviewed to calculate the IPA, IL and IW using the Elbahnasy method. A systematic review was also conducted for all English language articles between January 2000 and April 2018, reporting on the impact of pelvicaliceal anatomy on RIRS. A total of 108 patients with LPS were included with a male to female ratio of 2:3 and a mean age of 54.7 years. The mean lower pole stone size was 9.3 mm (range 3–29 mm) and 102/108 (94.4%) patients were stone free (SF) at the end of their procedure. While steep IPA (< 30°), operative time duration and larger stone size were significant predictors of failure, the placement of ureteric access sheath, IW and IL did not influence treatment outcomes. Six studies (460 patients) met the inclusion criteria for our review. The IPA, IW, IL for failure ranged from 26° to 38°, 5.5–7 mm and 24–34 mm, respectively. The SFR ranged from 78 to 88% with a metaanalysis showing IPA as the most important predictor of treatment outcomes for LPS. Infundibular pelvic angle seems to be the most important predictor for the treatment of LPS using RIRS. Pelvicalyceal anatomy in conjunction with stone size and hardness seem to dictate the success, and decisions on the type of surgical interventions should reflect this.

## Introduction

With an increase in the incidence of stone disease over the last 2 decades, there has been a corresponding rise in the surgical procedures undertaken for it [1–3]. Majority of stones in the kidney are located in the lower pole calyx [4]. Treatment of stones in the lower calyx includes shockwave lithotripsy (SWL), retrograde intrarenal surgery (RIRS) and percutaneous nephrolithotomy (PCNL) [4]. Stone treatment in the lower pole is less successful compared to treatment elsewhere in the kidney [5]. The risk of stone formation seems to be associated with a large calyceal volume and narrow infundibulum [6]. The impact of renal anatomy while treating lower pole renal stones (LPS) with SWL is well established, however, this is less well known and poorly evidenced with RIRS.

Elbahnasy documented a method for measuring the infundibular pelvic angle (IPA) and looked to correlate its effect with regards to treatment success with SWL [7]. Stone clearance rates for SWL in the lower pole are inferior to interpolar or upper pole calyces, with clearance rates ranging between 24.7 and 57.8% [8, 9]. Increasing stone size in the lower pole has also been identified with a worse stone free rate (SFR) with SWL [10]. IPA, infundibular length (IL) and infundibular width (IW) have all been described in relationship to the treatment success of SWL [11]. Although this association has been descried in RIRS for accessing the lower pole, the clinical data on treatment outcome is sparse [12–20].

We wanted to look at the role of lower pole anatomy (infundibular pelvic angle, infundibular length and infundibular width) to study clinical outcomes in patients treated for lower pole stones (LPS). We also perform a review of literature to look at the other published papers which study the influence of pelvicalyceal anatomy on success with RIRS.

## Methods

### The results of our data

Data were prospectively collected over a period of 3 years (June 2013–June 2016) for all patients who underwent RIRS for LPS, and the imaging (Computer tomography (CT) scans and retrograde pyelogram (RPG) images) were then retrospectively reviewed for patients who underwent RIRS for stones in the lower pole. The IPA, IL and IW were calculated using CT or X-ray calibrated RPG, and where imaging was unclear or unavailable then these patients were excluded. Measurement of the IPA was completed using the Elbahnasy [7] method.

A semi rigid ureteroscopy was performed over a working guidewire up to the pelvi-ureteric junction (PUJ) or as far proximally as safely achievable, which helped in the calibration of the ureter to judge whether a ureteral access sheath (UAS) could be inserted. If the ureter was judged to be tight and it was felt that the UAS could not be safely inserted, the flexible ureteroscope (fURS) was inserted radiologically over a safety wire. Flexible ureteroscopy was performed under general anaesthesia with Flex X2 (Karl Storz Endoscopy (UK) Ltd., Slough, UK) with a Holmium YAG laser, Lumenis (UK) Ltd., Elstree, UK) using a 272-μm laser fiber (Lumenis, Inc.) [21]. For large stones and where multiple passes of the scope was anticipated, if feasible a 9.5Fr/11.5Fr or a 12Fr/14Fr Cook Flexor UAS (Cook Medical, USA) was used and placed just below the pelviureteric junction (PUJ). Stone fragments were retrieved actively with a Cook Ngage^**®**^ nitinol stone extractor (Cook Medical, USA) and sent for biochemical analysis. Stone-free rate (SFR) was defined using a combination of being endoscopically stone free and radiologically stone free (defined as fragments ≤ 2 mm) on follow-up imaging [4]. The follow-up imaging was a mixture of plain X-ray for radiopaque stones or ultrasound (USS) for radiolucent stones with occasional non-contrast CT scan, done 2–3 months post-ureteroscopy.

### Review of the literature

#### Inclusion criteria

1. Studies reporting on RIRS for LPS with information on pelvicalyceal anatomy (IPA, IW and IL).
2. Articles written in English language with patients of all age groups.

#### Exclusion criteria

1. Case reports and review articles.
2. Animal and simulation studies.

All studies reporting on RIRS and the impact of pelvicalyceal anatomy on the treatment outcomes were identified between January 2000 and April 2018 using MEDLINE, EMBASE, CINAHL, Cochrane library, Clinicaltrials.gov, Google Scholar and Individual urological journals for all English language articles. The search terms used in conjunction with each other included: “retrograde intrarenal surgery”, “RIRS”, “retrograde ureteroscopy” “ureteroscopy”, “URS”, “ureterorenoscopy”, “pelvicalyceal anatomy”, “calculi”, “stone”, “infundibular width”, “infundibular height”, “pelvicalyceal angle” and “infundibular pelvic angle”. Two reviewers (SS and LH) independently identified all studies and any discrepancy was resolved by consensus with the senior author (BKS).

Statistical analysis was performed using SPSS version 24 [23]. Independent samples *t* test was used for continuous data and Chi squared test for dichotomous data. Results of statistical analysis presented as *p* values with 95% confidence intervals for *t* tests and *p* values alone for Chi squared test.

## Results

### Results of our series

Between June 2013 and June 2016, a total of 108 patients with LPS were included. The male to female ratio was 2:3 with a mean age of 54.7 years (range 7–86 years). Of these LPS stones, 101 had unilateral and 7 had bilateral RIRS procedures.

The mean lower pole stone size was 9.3 mm (range 3–29 mm). A ureteric access sheath (UAS) was placed in 59 (56.2%) patients with a post-operative ureteric stent (with or without a string attached) in 100 (92.6%) patients. 102/108 (94.4%) patients were stone free (SF) at the end of their procedure. The placement of a ureteric access sheath had no significant impact on stone free rate (SFR) (*p* = 0.53).

Comparison was made between SF and non-stone free (NSF) patients (See Table 1, Fig. 2). Steep IPA angle was found to be a significant predictor of failure. The greatest percentage of NSF patients had an IPA < 30° (*p* < 0.05, 95% CI [31.8, 51.9]) (Table 1). These patients also had larger stones and longer operative time duration. There was, however, no significant difference in IL (*p* = 0.65, 95% CI [5.4, − 3.4]) and IW (*p* = 0.26, 95% CI [2.9, 0.8]) between these two groups. Two complications were recorded in the SF group, including post-operative catheterisation (Clavien I; IPA 30.6, IL − 28.7 mm, IW 9.8 mm) and respiratory sepsis (Clavien II; IPA 34.4, IL − 22.6, IW − 9.1 mm). There were no reported complications in the NSF group.

**Table 1:** Lower pole stone characteristics

*T* test	SF	NSF	*p*	95% CI
IPA (°)	38.1 ± 6.8	32.4 ± 6.0	**0.05**	0.0 to 11.3
IL (mm)	24.8 ± 5.1	23.8 ± 6.1	0.65	5.4 to − 3.4
IW (mm)	8.1 ± 2.2	7.0 ± 1.5	0.26	2.9 to 0.8
Operative time	47.9 ± 26.7	74.7 ± 35.7	**0.02**	11.6 to 3.7
Largest stone diameter (mm)	9.1 ± 5.1	14.8 ± 5.7	**0.009**	2.2 to 1.5

Statistically significant values are in bold (*p* < 0.05)

*IPA* infundibular pelvic angle, *IL* infundibular length, *IW* infundibular width, *SF* stone free, *NSF* not stone free

### Literature review

**Fig. 1:**
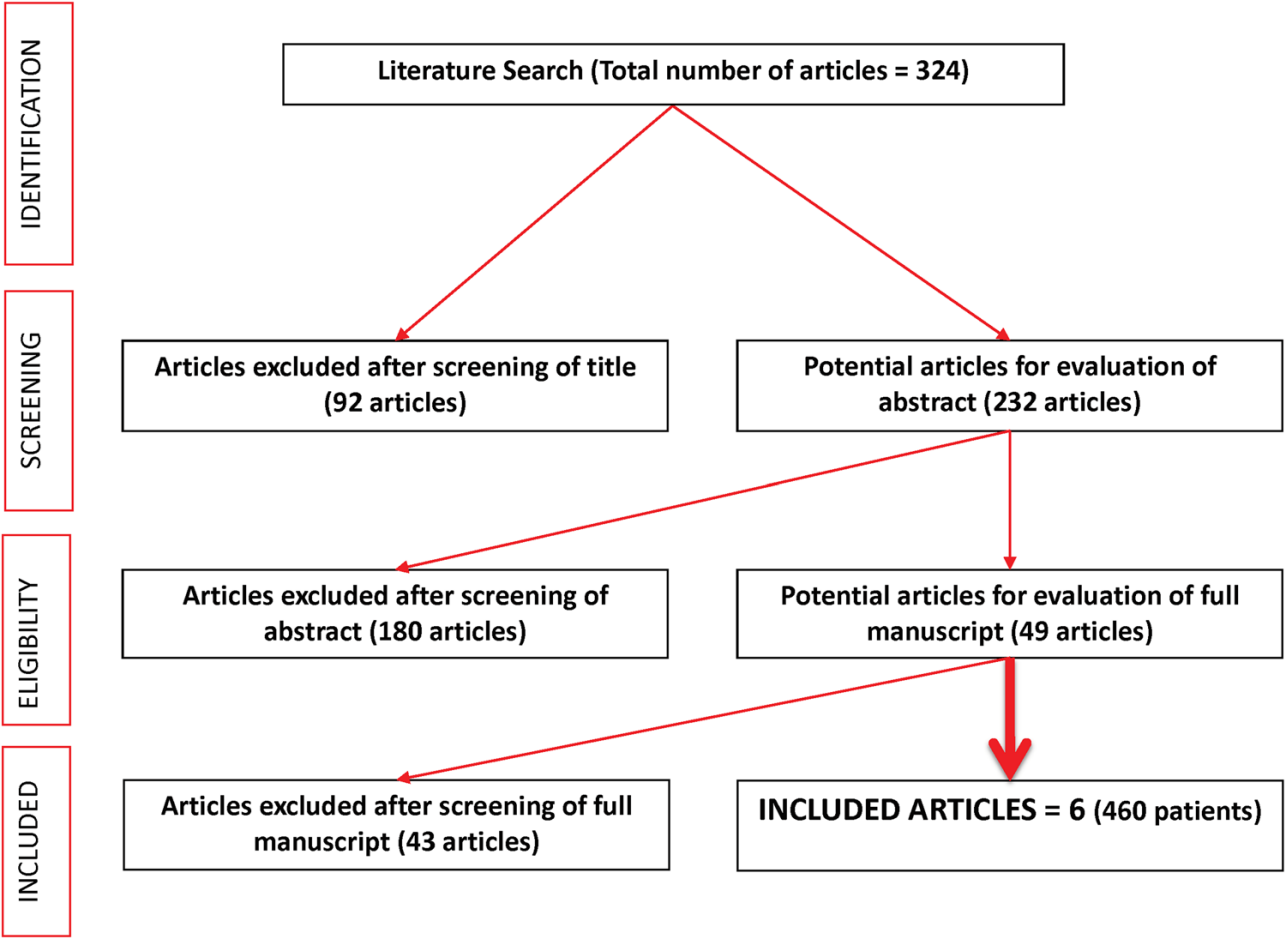
PRISMA flowchart of included studies

Between January 2000 and April 2018, a total of 232 abstracts were reviewed. Of these, 6 studies met the inclusion criteria and reported on a total of 460 patients (Fig. 1).

**Fig. 2:**
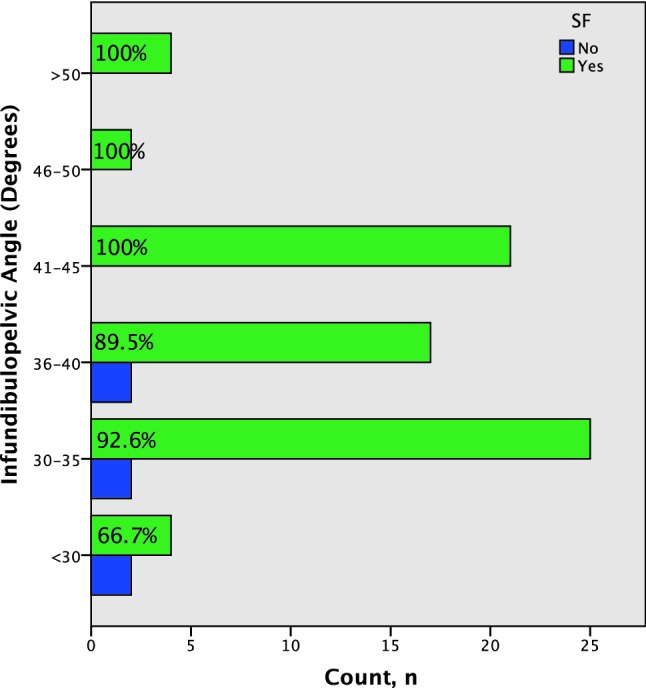
Correlation of stone free rate (SFR) with infundibulopelvic angle (IPA)

Of these 460 patients, 79.5% (range 78–88.3%) had a successful procedure and were stone free. Four papers described stone free as no residual fragments [12, 13, 16, 17], while the remaining two studies describing stone free as < 2 mm fragments [20] and < 4 mm fragments [14], respectively (Table 2).

**Table 2:** Previous studies reporting on the pelvicalyceal angle

Author	Year	Number of patients	Success	Scope	Imaging for measurement	Size of stones (mm) (stone free)	Size of stones (mm) (not stone free)	Definition of success
Geavlete [20]	2008	47	34 (70.2%)	Storz 7.5 Fr	RPG		Mean = 8.3	< 2 mm fragments
Resorlu [14]	2012	67	54 (80.6%)	7.5 Fr Karl storz/Olympus 8.4 Fr	IVU		Mean = 16.88	< 4 mm
Jensen [16]	2014	111	87 (88.3%)	Flex-X2	RPG and IVU		Mean = 7.47	No residual fragments
Kilicarslan	2015	36	28 (77.8%)	Flex-X2	IVU	Median—10 mm	Median—12 mm	No residual fragments
Inoue [17]	2015	67	55 (82.1%)	Flex-X2	IVU	Mean—26.6 mm	Mean—29.1 mm	No residual fragments
Sarı [12]	2017	132	103 (78%)	Flex-X2	IVU	Mean—10 mm		No residual fragments

*RPG* retrograde pyelogram, *IVU* intravenous urogram

All six studies reported on infundibular pelvic angle and infundibular length, with five also reporting on infundibular width [12–14, 16, 17]. When measuring the infundibular pelvic angle measurements were taken using either the intravenous urogram (IVU) or RPG. Three studies along with our study used the Elbahnasy method to calculate infundibular pelvic angle and reported on the mean infundibular pelvic angle, length and width in both successful and unsuccessful procedures [14, 16, 17] (Table 2). They examined the pelvicalyceal anatomy and the chance of successful stone treatment (Table 3).

**Table 3:** All studies which mentioned successful and unsuccessful lower pole stone treatment and calculated the pelvicalyceal anatomy using the Elbahnasy [7] method

Year	Author	Number of patients	Successful procedure			Unsuccessful procedure		
			IPA (°)	IL (mm)	IW (mm)	IPA (°)	IL (mm)	IW (mm)
2015	Inoue [17]	67	44.0	27.2	8.8	26.7	33.6	6.8
2014	Jessen [16]	111	47.3	22.5	6.0	36.5	28.3	6.2
2012	Resorlu [14]	67	49.4	26.8	5.8	37.6	28.2	5.6
2018	Current study	108	38.1	24.8	8.1	32.4	23.8	7.0

*IPA* infundibular pelvic angle, *IL* infundibular length, *IW* infundibular width

**Fig. 3:**
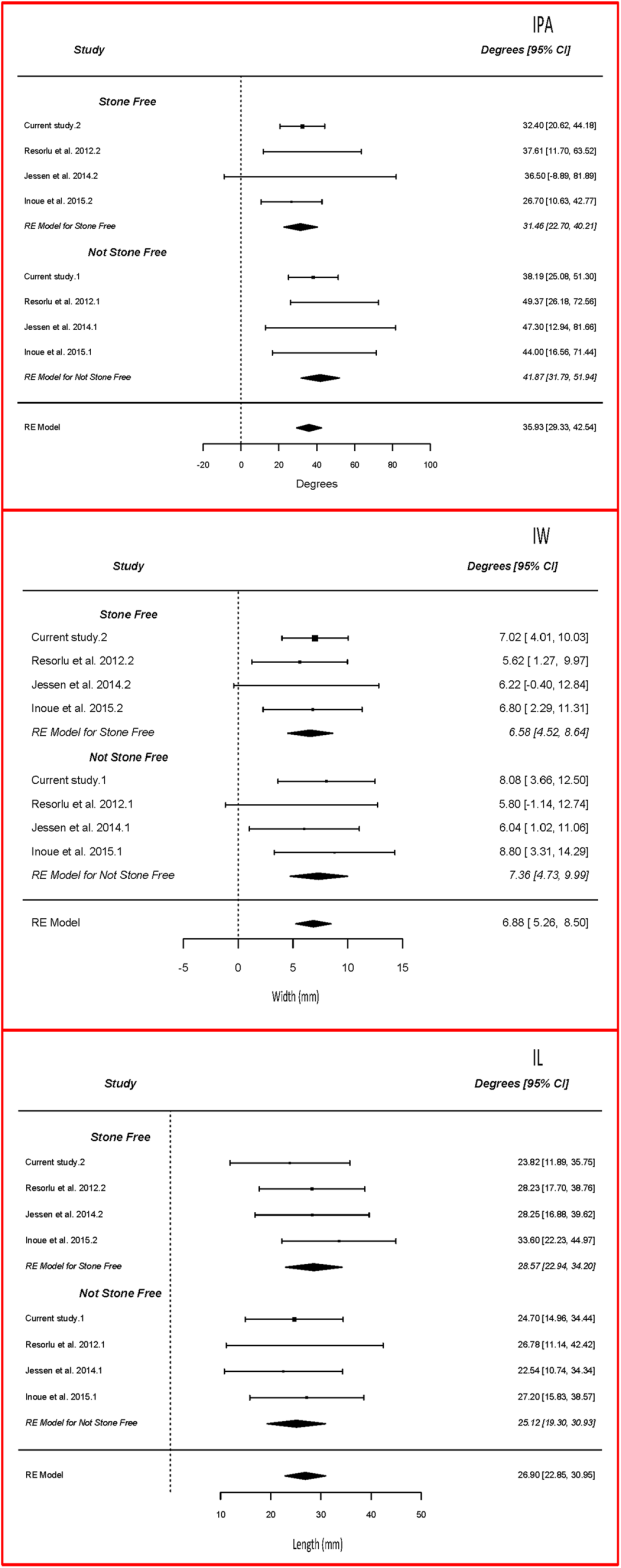
Pelvicalyceal anatomy (IPA, IW, IL) meta-analysis

In our patient cohort most of our NSF patients had an IPA < 30° (Tables 1, 3). We performed a meta-analysis for pelvicalyceal anatomy between SF and NSF patients (Fig. 3). Across the meta-analyses the average IPA was notably smaller in the NSF group (31.5°, *p* < 0.05, 95% CI [22.7, 40.2]) when compared to SF group (41.9°, *p* < 0.05, 95% CI [31.8, 51.9]). However, no significant difference (*p* = 0.126, 95% CI [2.95, 23.8]) in IPA was identified between the two groups. On subgroup analysis, the IL (*p* = 0.40, 95% CI [− 11.6, 4.60]) and IW (*p* = 0.65, 95% CI [− 2.56, 4.12]) showed no significant difference between the SF and NSF groups with data suggesting that IPA was the most important predictor of treatment outcome for stones in the lower pole (Figs. 2, 3).

## Discussion

### Meaning of our study

Based on our data and the review of literature, the infundibular pelvic angle seems to be the most important determinant of lower pole stone treatment, and a success rate of over 94% can be achieved for LPS. Patients who were not stone free also had larger stones and consequently had longer operative time duration.

### Role of infundibular pelvic angle on SFR for LPS

The IPA across the four studies for successful and unsuccessful procedures ranged from 38°–50° to 26°–38°, respectively. Although the meta-analyses did not identify a significant difference in the IPA between the NSF and SF groups, the wide confidence intervals indicate the studies were likely underpowered and, therefore, may represent a type 2 error. Various methods have been used for measuring anatomy relating to the lower pole calyx [7, 24]. Manikandan [19] examined collecting systems using three methods for measuring the IPA, for a stone bearing and contralateral non-stone bearing kidney and found that the only statistically significant difference in IPA was identified using the Elbahnasy method [7]. Our results show the importance of IPA and is comparable to other series [17] and confirms that with a cut-off of < 30° there was a statistically significant difference in SFR [16, 17], although one study has suggested a cut-off of 45° as significant [14].

### Role of infundibular width on SFR for LPS

The infundibular width across the four studies for successful and unsuccessful procedures ranged from 6–9 mm to 5.5–7 mm, respectively [14, 16, 17]. For LPS treated with SWL an infundibular width < 5 mm has been associated with a lower SFR, with stone clearance of 47% and 83% below and above this cut-off (*p* = 0.0001) [13]. In our study, IW was not statistically significant, which has been mirrored in other studies too [14].

### Role of infundibular length on SFR for LPS

The infundibular length across the four studies for successful and unsuccessful procedures ranged from 22–27 mm to 24–34 mm, respectively. For LPS treated with SWL an infundibular length > 30 mm has been associated with a lower SFR, with stone clearance of 80% and 43% below and above this cut-off (*p* = 0.01) [13]. In our study IL was not statistically significant although other authors have shown IL to be significant [16]. In an attempt to refine parameters to determine operative success they looked at cut off values and found that IL > 27 mm was identified to have a significant effect on the SF status.

### Role of stone size on SFR for LPS

When Elbahnasy compared all three treatment modalities (SWL, PCNL and RIRS) he was able to demonstrate that a stone size > 1 cm had a decreased SFR across all groups [7]. When looking at the impact of URS for LPS, increase in the mean stone size had a significant impact on SFR in our study, which has been confirmed by other studies [14, 16].

### Role of stone composition on SFR for LPS

Stone composition also seems to affect the SF status for LPS. Patients with stones composed of brushite, cystine and calcium oxalate monohydrate have been shown to have a lower success rate when treated with SWL [11]. Brushite stones have also shown to have a statistically significant effect on SF status with RIRS [16]. Hounsfield units (HU) have been used as a surrogate marker for the hardness of stone and a value > 1000 HU seems to affect the SFR with RIRS [17]. The number of stones in the LP has also been suggested to play a role wherein patients with multiple calculi had a lower SFR [15].

### Role of other factors on SFR for LPS

Consideration may also be taken to previous operative intervention such as previous PCNL, which may predict a lower SF status for LPS treated with RIRS [15], although a prior history of SWL did not have a significant impact on SFR [14]. Martin and colleagues also looked at additional intra-operative factors including surgeon experience, ureteral access sheath, and preoperative stents and were not able to show any significant difference in SFR in patients treated for lower and non-lower pole stones [15].

### Strengths, limitations and areas of future research

We have adhered to the methodological approach of Cochrane guidelines [25] and the PRISMA checklist [26], however, the study is limited by the available literature which is retrospective cohort studies. Despite having limited data, we performed a meta-analysis to look at the IPA, IL and IW and its relationship to the success and failure regarding SFR.

All of the available studies were retrospective in nature and did not have a standardised method of measuring the pelvicalyceal anatomy. Although there are plenty of studies comparing different treatment options for LPS, majority of them do not look at the pelvicalyceal anatomy which predicts the success of these treatments. Future studies should include this when comparing LPS treatment, to give clinicians an algorithm of individualised treatment based on the likelihood of success for a given stone. Finally, it is about time we agreed on a universally agreed imaging modality and definition of SFR which would help to draw meaningful conclusions when comparing different treatment modalities [22].

Steep IPA also seems to be a risk factor for flexible ureteroscope damage and complicated post-operative course [27]. Although our review shows a good SFR for LPS, information on pelvicalyceal anatomy might prove to be useful when treating stones with unfavourable anatomy especially when they are large and have a hard composition. While IW and IL may not be as important, IPA seems to be important (< 30°) and in these cases an alternate treatment option such as minimally invasive PCNL procedure could be considered [28, 29]. This is especially important for informed decision making with the patients, when counselling them for the treatment success for LPS and discussing alternate treatment options.

## Conclusion

Retrograde intrarenal surgery is an effective treatment option for the management of lower pole stones. Infundibular pelvic angle seems to be the most important predictor for being stone free. Pelvicalyceal anatomy in conjunction with stone size and hardness seem to dictate the success of RIRS for lower pole stones and decisions on the type of surgical interventions should reflect this.
